# A riboflavin transporter deficiency presenting as pure red cell aplasia: a pediatric case report

**DOI:** 10.3389/fped.2024.1391245

**Published:** 2024-04-17

**Authors:** Jingying Cheng, Jiafeng Yao, Shasha Zhao, Lingling Fu, Liqiang Zhang, Jin Jiang

**Affiliations:** Department of Hematology, National Center for Children’s Health, Beijing Children’s Hospital, Capital Medical University, Beijing, China

**Keywords:** riboflavin, pure red cell aplasia, genetic testing, *SLC52A2*, children

## Abstract

**Introduction:**

Riboflavin transporter deficiency (RTD) is a rare genetic disorder that affects riboflavin transport, leading to impaired red blood cell production and resulting in pure red cell aplasia. Recognizing and understanding its clinical manifestations, diagnosis, and management is important.

**Case presentation:**

A 2-year-old patient presented with pure red cell aplasia as the primary symptom of RTD. After confirming the diagnosis, rapid reversal of anemia was achieved after high-dose riboflavin treatment.

**Conclusion:**

RTD often has an insidious onset, and neurological symptoms appear gradually as the disease progresses, making it prone to misdiagnosis. Genetic testing and bone marrow biopsy can confirm the diagnosis.

## Introduction

Riboflavin transporter deficiency (RTD), also known as Brown-Vialetto-Van Laere syndrome, is a neurological disorder caused by pathogenic variants in the riboflavin transporter protein genes *SLC52A2* (encoding RFVT2) and *SLC52A3* (encoding RFVT3). The most common manifestations are sensorineural hearing loss (SNHL), peripheral neuropathy, respiratory insufficiency, and medullary paralysis (1, 2). Reports of hematological manifestations as primary symptoms are extremely rare. Here, we report a case of RTD with pure red cell aplasia as the initial symptom. This study aimed to enhance our understanding of hematological manifestations and non-targeted metabolomic analysis in the treatment of RTD to achieve early detection, diagnosis, and treatment.

## Case report

When the 2-year-old patient was 2 months old, he was brought to the Hematology Department of Beijing Children's Hospital outpatient clinic due to “pale complexion for over 2 months”. Initial blood tests revealed the following results: red blood cells (RBC), 1.02 × 10^12^/L (Normal 3.5–5.6); hemoglobin (Hb), 29 g/L (Normal 99–196); hematocrit, 9.2% (Normal 29–57); mean corpuscular volume, 90.2 fl (Normal 73–105); MCH, 28.4 pg (Normal 24–37); mean corpuscular Hb concentration, 315 g/L (Normal 305–361); Reticulocyte %, 0.48% (Normal 0.5–2.5); Reticulocyte absolute value, 4.9 × 10^9^/L (Normal 22–139). White blood cell and platelet counts were within normal ranges. The patient received blood transfusions to correct the anemia. The patient had normal development until 6 months old, followed by delayed motor development and sensory ataxia. At 1 year old, the patient had difficulty grasping, sitting, and standing without support and did not crawl. Due to the young age of the patient, some symptoms cannot be self-reported, and cannot cooperate with auditory and visual examinations. The mother had a history of preexcitation syndrome and was prone to syncope. Mild anemia developed during pregnancy with unknown etiology. A physical examination of the patient revealed severe anemia, with no enlarged superficial lymph nodes, liver, or spleen. Bilirubin and anemia-related indicators were normal, and the patient was negative for autoimmune antibodies. No pathogenic variants were found in the genetic screening of a blood system panel. Based on the onset of symptoms within the first year of life and test results, a diagnosis of pure red cell aplasia was considered. Due to the young age of the patient and the potential impact of hormones on growth and development, symptomatic blood transfusion therapy was temporarily initiated, requiring monthly transfusions when Hb levels dropped below 70 g/L. At 6 months old, the patient experienced breath-holding spells accompanied by cyanosis of the face and lips after crying. These lasted for a few seconds and resolved spontaneously. Occasionally, episodes of limb stiffness and termor manifested, which improved after sedative therapy. No fever or infection was observed during the interictal period and no abnormalities were observed in the electroencephalogram at that time.

**Figure 1:**
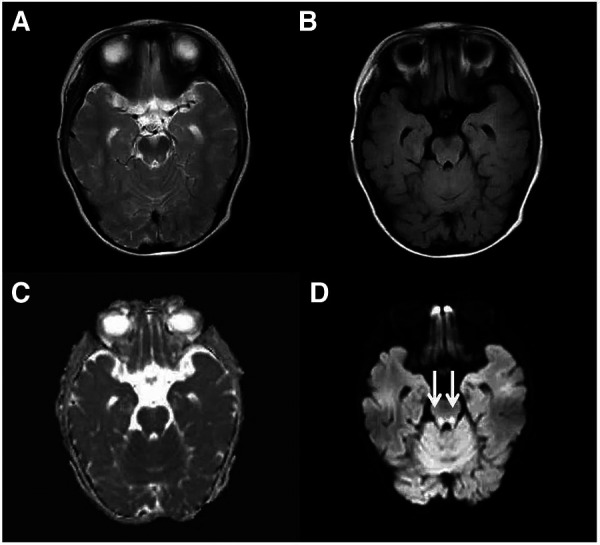
Cranial MRI at the level of the midbrain. An axial T2-weighted (**A**), an axial FLAIR (**B**), a dADC (**C**) and a DWI (**D**) scans are shown and abnormally high signals in DWI in the midbrain can be seen (white arrow).

**Figure 2:**
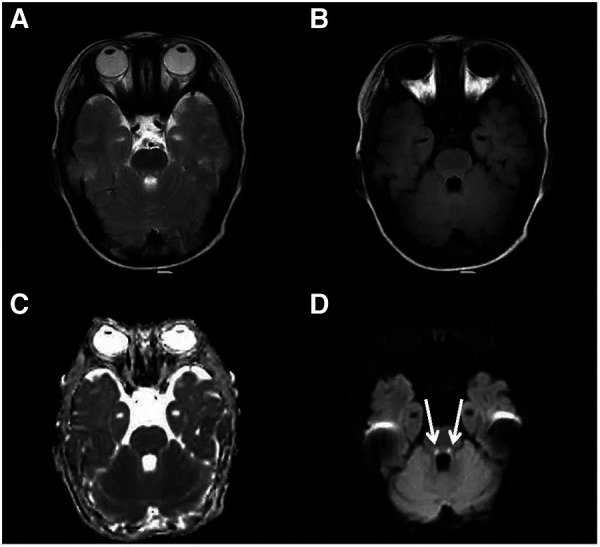
Cranial MRI at the level of the dorsal pons. An axial T2-weighted (**A**), an axial FLAIR (**B**), a dADC (**C**) and a DWI (**D**) scans are shown and abnormally high signals in DWI in the dorsal pons can be seen (white arrow).

**Figure 3:**
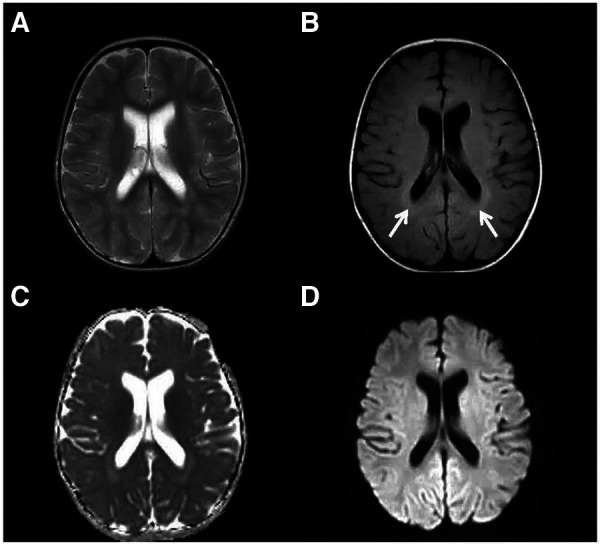
Elevated T2 FLAIR signal in the periventricular white matter. An axial T2-weighted (**A**), an axial FLAIR (**B**), a dADC (**C**) and a DWI (**D**) scans are shown and slightly elevated T2 FLAIR signal in the periventricular white matter can be seen (white arrow).

During the evaluation of the 1-year-old patient, a follow-up bone marrow morphological examination revealed active bone marrow hyperplasia with relatively normal erythroid proliferation, mainly consisting of intermediate and young RBCs. The granulocyte-erythrocyte ratio was slightly higher, and the morphology was generally normal. Early erythroid cells accounted for 1% (Normal 0–5.8), intermediate erythroid cells for 8% (Normal 5–34), and late erythroid cells for 2.5% (Normal 1.6–21.5). A bone marrow biopsy showed reduced erythroid proliferation. Therefore, pure red cell aplasia was not ruled out. Chromosomal karyotyping revealed 46, XY, del(5)(q33)[2]/46, XY[18]. Comprehensive testing for myelodysplastic syndrome and myeloid leukemia-related genes did not reveal any pathogenic variants. Based on the patient's medical history, symptoms, signs, and auxiliary examinations, pure red cell aplasia was diagnosed. Therefore, starting on October 1, 2022, the patient was treated with prednisone 10 mg BID for 2 months, followed by methylprednisolone 16 mg QD for 1 month. On December 21, 2022, oral cyclosporine was added, and methylprednisolone was discontinued after 1 month. The cyclosporine treatment lasted 6 months, but the treatment was ineffective, and the patient still required transfusions. At the age of 22 months, the patient developed progressive upper limb weakness, especially in the hands. The patient was subsequently examined at the neurology outpatient departments of Beijing Children's Hospital and Beijing Xuanwu Hospital. Cranial magnetic resonance imaging showed bilateral symmetric punctate high signals in the midbrain and dorsal pons in T2W imaging, along with high signals in DWI and low signals in ADC. Additionally, there was bilateral ventricular dilatation and slightly elevated T2 FLAIR signal in the periventricular white matter (Figures 1–3). Magnetic resonance imaging of the cervical spine showed patchy, long T1 and T2 signals in the spinal cord. Genetic metabolic screening revealed no significant abnormalities. Whole-exome sequencing revealed compound heterozygous variants in *SLC52A2*: c.588C > G (variant inherited from the father, heterozygous variant) and c.593G > A (variant inherited from the mother, heterozygous variant). Previous genetic tests did not include analysis of *SLC52A2* genes. According to the ACMG guidelines, the variant c.588C > G is of uncertain clinical significance (PM1, PM2, PP3_Supporting), whereas variant c.593G > A is suspected to be pathogenic (PVS1_VeryStrong, PM2). The REVEL protein function damage prediction for c.588C > G is 0.718, and for c.593G > A is 0. The patient was started on oral riboflavin (vitamin B2) at a dose of 10 mg/d (0.8 mg/kg/d) on July 17, 2023, gradually increasing to 75 mg/d (6.25 mg/kg/d). Consequently, the vitamin B2 blood concentration increased from 8.18 ug/L to 11 ug/L. After one week of treatment, the Hb level increased to 116 g/L and fluctuated between 126 and 137 g/L over the next two weeks. Upper limb muscle strength improved from grade 2 to grade 3+, with the ability to perform simple grasping. Unfortunately, nystagmus developed, indicating deterioration of the patient's condition. Due to difficulty in sedating the patient, electromyography could not be completed. The patient is currently under follow-up in the hematology and neurology outpatient departments.

## Discussion

RTD is an autosomal recessive progressive neurological disorder characterized by early-onset SNHL, bulbar palsy, optic atrophy, severe diffuse muscle weakness, and atrophy of the upper and lower limbs and axial muscles, leading to respiratory insufficiency (1, 2). Before diagnosis, patients may experience optic atrophy, sleep apnea, breath-holding spells, or swallowing difficulties (3). RTD is mainly caused by genetic variants in *SLC52A2* or *SLC52A3*, which results in riboflavin transporter function deficiencies (4, 5). These disorders are referred to as RTD2 and RTD3, respectively. RTD3 typically presents with more generalized weakness. In approximately 92% of RFVT2-deficient patients, the upper limb muscles are more affected than the lower limb muscles, and most patients experience weakness in the shoulder girdle and distal hand muscles (6). These findings are consistent with those of the male patient described here, who presented with breath-holding spells at six months of age, followed by delayed motor development and tremors in both hands at 22 months old.

*SLC52A2* encodes a transmembrane protein (RFVT2) that mediates the cellular uptake of riboflavin. The most common pathogenic variants are missense mutations (94.4%), followed by nonsense (2.4%), splice-site (0.8%), deletion (1.6%), and insertion mutations (0.8%) (7, 8). In this case, the patient had compound heterozygous pathogenic variants in *SLC52A2*. The c.588C > G variant is a novel pathogenic variant that has not been previously reported. The c.593G > A variant was reported in a 2019 study discussing the etiology of pediatric SNHL (9).

RTDs have a low incidence rate and significant clinical heterogeneity. Patients often present with neurological symptoms or deafness, and approximately 70% of individuals have an abnormal acylcarnitine profile before riboflavin supplementation (10). Due to impaired iron absorption resulting from riboflavin deficiency, some patients may experience mild-to-moderate decreases in RBC counts and/or Hb levels. Riboflavin-responsive macrocytic anemia was first reported in a patient with RTD in 2020 (3). However, rapid correction of anemia was achieved with high-dose riboflavin treatment. Subsequently, Pillai et al. reported the case of a 2-year-old boy diagnosed with RTD who had a history of seizures and breath-holding spells at 21 months of age. Bone marrow biopsy revealed active bone marrow proliferation with significantly delayed RBC maturation and maturation arrest. Subsequently, the patient developed ataxia and developmental regression. After two weeks of treatment with riboflavin, the patient's hematological abnormalities completely resolved, and the neurological symptoms eventually improved (11). In 2021, a patient presenting with pure red cell aplastic anemia was reported in China and was found to have novel *SLC52A2* pathogenic variants. The patient's symptoms significantly improved with supplementation of low-dose riboflavin (20 mg/d) (12).

Because riboflavin must be obtained through intestinal absorption, extensive research has found that oral riboflavin supplements can effectively alleviate the clinical symptoms of patients with RTD. However, if irreversible damage has already occurred, the therapeutic effect is minimal. Therefore, early detection, definitive diagnosis, and initiation of riboflavin treatment are crucial. The literature reports a dosage range of oral riboflavin from 7 to 60 mg/kg/d, with no reports of death and few adverse reactions (13–16). After treatment, approximately 60% and 80% of patients with RFVT2 and RFVT3 defects, respectively, showed improvement in clinical symptoms, with most patients with RTD experiencing improvement in symptoms within days to months (17).

## Summary

RTD often presents with an insidious onset and lacks obvious abnormalities in cranial imaging changes, making it challenging to differentiate from various neurodegenerative diseases. Although RTD is mainly characterized by early onset SNHL and optic nerve atrophy, the age of onset is young, and the patients usually cannot accurately describe visual and auditory abnormalities (18). Moreover, identifying auditory neuropathy by conventional hearing screening is generally difficult, leading to missed diagnoses (19, 20). Currently, molecular biological diagnosis and genetic testing are the main methods used for diagnosing RTD. Therefore, physicians need to enhance their understanding of the clinical manifestations of RTD and suggest a more comprehensive evaluation, along with early genetic diagnosis, for patients suspected of having RTD or patients presenting with anemia/red cell aplasia without other features of RTD. If a physician suspects RTD and patients have severe symptoms, empirical riboflavin treatment can be started immediately without waiting for molecular diagnosis results because the treatment is relatively safe (21).

Owing to the irreversibility of nerve damage, the timing of riboflavin treatment for RTD is crucial, and early initiation of treatment is key. Therefore, performing *SLC52A2* genetic testing for patients suspected of having RTD should be performed as soon as possible. Applying these tests to neonatal screening should be considered to achieve rapid diagnosis and early treatment to minimize hematological and neurological damage.

## Data Availability

The raw data supporting the conclusions of this article will be made available by the authors, without undue reservation.
